# Chemopreventive and Therapeutic Efficacy of *Cinnamomum zeylanicum* L. Bark in Experimental Breast Carcinoma: Mechanistic In Vivo and In Vitro Analyses

**DOI:** 10.3390/molecules25061399

**Published:** 2020-03-19

**Authors:** Peter Kubatka, Martin Kello, Karol Kajo, Marek Samec, Karin Jasek, Desanka Vybohova, Sona Uramova, Alena Liskova, Vladimira Sadlonova, Lenka Koklesova, Radovan Murin, Marian Adamkov, Karel Smejkal, Emil Svajdlenka, Peter Solar, Samson Mathews Samuel, Monika Kassayova, Taeg Kyu Kwon, Pavol Zubor, Martin Pec, Jan Danko, Dietrich Büsselberg, Jan Mojzis

**Affiliations:** 1 Department of Medical Biology, Jessenius Faculty of Medicine, Comenius University in Bratislava, 03601 Martin, Slovakia; martin.pec@uniba.sk; 2 Division of Oncology, Biomedical Center Martin, Comenius University in Bratislava, Jessenius Faculty of Medicine, 036 01 Martin, Slovakia; Karin.Jasek@uniba.sk (K.J.); sona.uramova@uniba.sk (S.U.); 3 Department of Pharmacology, Faculty of Medicine, P. J. Šafarik University, 040 11 Košice, Slovakia; kellomartin@yahoo.com; 4 St. Elisabeth Oncology Institute, Department of Pathology, 812 50 Bratislava, Slovakia; kkajo@ousa.sk; 5 Biomedical Research Center, Slovak Academy of Sciences, 845 05 Bratislava, Slovakia; 6 Department of Obstetrics and Gynecology, Jessenius Faculty of Medicine, Comenius University in Bratislava, 036 01 Martin, Slovakia; marek.samec@uniba.sk (M.S.); alenka.liskova@gmail.com (A.L.); koklesova.lenka@gmail.com (L.K.); jan.danko@uniba.sk (J.D.); 7 Department of Anatomy, Jessenius Faculty of Medicine, Comenius University in Bratislava, 036 01 Martin, Slovakia; desanka.vybohova@jfmed.uniba.sk; 8 Department of Microbiology and Immunology, Jessenius Faculty of Medicine, Comenius University in Bratislava, 036 01 Martin, Slovakia; vladimira.sadlonova@uniba.sk; 9 Department of Biochemistry, Jessenius Faculty of Medicine, Comenius University in Bratislava, 036 01 Martin, Slovakia; radovan.murin@jfmed.uniba.sk; 10 Department of Histology and Embryology, Jessenius Faculty of Medicine, Comenius University in Bratislava, 036 01 Martin, Slovakia; marian.adamkov@uniba.sk; 11 Department of Natural Drugs, Faculty of Pharmacy, University of Veterinary and Pharmaceutical Sciences, 612 42 Brno, Czech Republic; karel.mejkal@post.cz (K.S.); svajdlenkae@fvu.cz (E.S.); 12 Department of Medical Biology, Faculty of Medicine, P. J. Safarik University, 04011 Kosice, Slovakia; solarpeter@yahoo.com; 13 Weill Cornell Medicine in Qatar, Qatar Foundation-Education City, 24144 Doha, Qatar; sms2016@qatar-med.cornell.edu; 14 Department of Animal Physiology, Institute of Biology and Ecology, Faculty of Science, P. J. Šafarik University, 04001 Košice, Slovakia; monika.kassayova@upjs.sk; 15 Department of Immunology and School of Medicine, Keimyung University, Dalseo-Gu, 42601 Daegu, Korea; kwontk@dsmc.or.kr; 16 OBGY Health & Care, Ltd., 01001 Zilina, Slovakia; prof.pavol.zubor@gmail.com

**Keywords:** apoptosis, cancer stem cells, cell proliferation, *Cinnamomum zeylanicum*, epigenetics, mammary carcinogenesis, MCF-7 cells, MDA-MB-231 cells, mouse, preventive medicine, rat

## Abstract

Comprehensive oncology research suggests an important role of phytochemicals or whole plant foods in the modulation of signaling pathways associated with anticancer action. The goal of this study is to assess the anticancer activities of *Cinnamomum zeylanicum* L. using rat, mouse, and cell line breast carcinoma models. *C. zeylanicum* (as bark powder) was administered in the diet at two concentrations of 0.1% (w/w) and 1% (w/w) during the whole experiment in chemically induced rat mammary carcinomas and a syngeneic 4T1 mouse model. After autopsy, histopathological and molecular evaluations of mammary gland tumors in rodents were carried out. Moreover, in vitro analyses using MCF-7 and MDA-MB-231 cells were performed. The dominant metabolites present in the tested *C. zeylanicum* essential oil (with relative content over 1%) were cinnamaldehyde, cinnamaldehyde dimethyl acetal, cinnamyl acetate, eugenol, linalool, eucalyptol, limonene, o-cymol, and α-terpineol. The natural mixture of mentioned molecules demonstrated significant anticancer effects in our study. In the mouse model, *C. zeylanicum* at a higher dose (1%) significantly decreased tumor volume by 44% when compared to controls. In addition, treated tumors showed a significant dose-dependent decrease in mitotic activity index by 29% (0.1%) and 45.5% (1%) in comparison with the control group. In rats, *C. zeylanicum* in both doses significantly reduced the tumor incidence by 15.5% and non-significantly suppressed tumor frequency by more than 30% when compared to controls. An evaluation of the mechanism of anticancer action using valid oncological markers showed several positive changes after treatment with *C. zeylanicum*. Histopathological analysis of treated rat tumor specimens showed a significant decrease in the ratio of high-/low-grade carcinomas compared to controls. In treated rat carcinomas, we found caspase-3 and Bax expression increase. On the other hand, we observed a decrease in Bcl-2, Ki67, VEGF, and CD24 expressions and MDA levels. Assessment of epigenetic changes in rat tumor cells in vivo showed a significant decrease in lysine methylation status of H3K4m3 and H3K9m3 in the high-dose treated group, a dose-dependent increase in H4K16ac levels (H4K20m3 was not changed), down-regulations of miR21 and miR155 in low-dose cinnamon groups (miR22 and miR34a were not modulated), and significant reduction of the methylation status of two out of five gene promoters—*ATM* and *TIMP3* (*PITX2*, *RASSF1*, *PTEN* promoters were not changed). In vitro study confirmed results of animal studies, in that the essential oil of *C. zeylanicum* displayed significant anticancer efficacy in MCF-7 and MDA-MB-231 cells (using MTS, BrdU, cell cycle, annexin V/PI, caspase-3/7, Bcl-2, PARP, and mitochondrial membrane potential analyses). As a conclusion, *C. zeylanicum* L. showed chemopreventive and therapeutic activities in animal breast carcinoma models that were also significantly confirmed by mechanistic evaluations in vitro and in vivo.

## 1. Introduction

Extensive oncology (mainly preclinical) research has demonstrated that plant-derived substances, administered as both whole foods and isolated molecules, indisputably affect all stages of cancer disease including mammary carcinogenesis [1,2,3,4,5,6,7]. Phytochemicals have been documented as molecules that show significant antioxidant, anti-inflammatory, and immunomodulatory activity in vitro and in vivo. Moreover, many plant-derived substances have been proved to regulate the cell cycle, programmed cell death, angiogenesis, and activity of stem cells in cancer tissue and thus may suppress the growth and spread of malignant cells in organisms [8,9]. In addition, the tumor-suppressive potential of plant bioactive molecules also includes intensively discussed targeting of the aberrant epigenetic modifications particularly on the level of the global DNA hypomethylation status of the cells, hypermethylation of tumor suppressor gene promoters, histone post-translational modifications, and non-coding RNA (ncRNA)-linked multi-gene network modulations [10,11,12,13]. However, regarding clinical research, only a relatively limited number of epidemiological studies and meta-analyses have documented that regular (4–5 times a week) and long-term (several years) consumption of selected whole plant foods (not isolated molecules) is significantly associated with a decrease in cancer risk, including breast carcinoma (BC) [14,15,16,17].

The essential oil of *C. zeylanicum* L. bark (EOC) represents a lipophilic extract rich in some monoterpenoids (e.g., α-terpineol, caryophyllene, geraniol, phellandrene, borneol, carvacrol), sesquiterpenoids (e.g., limonene and linalool), with the main part formed by phenylpropanoids cinnamaldehyde, cinnamyl acetate, eugenol, and also simple aromatics such as benzaldehyde [18]. Together with relatively hydrophilic cinnamic acid, tannins, and some flavonoids, these secondary metabolites categorize *C. zeylanicum* bark amongst the spices and plant foods with the highest overall antioxidant capacity [19]. There are several preclinical studies pointing to the oncostatic potential of *C. zeylanicum* bark. *C. zeylanicum* showed significant cytotoxic and proapototic effects in *ras* active fibroblastic 5RP7 cells [20]. In another preclinical study, cinnamaldehyde has been documented as an antioxidant that reduced visfatin-induced breast cancer progression in vitro and in vivo [21]. The *C. zeylanicum* component 2-methoxycinnamaldehyde downregulated NF-κB binding activity, proliferative control involving programmed cell death (Bax/Bcl-2 increase), and topoisomerases I/II activities, and upregulated lysosomal vacuolation in human lung adenocarcinoma A549 cells in vitro and in vivo [22]. In the same cancer line, similar anticancer effects (inhibition of proliferation and apoptosis induction) were shown after cuminaldehyde treatment [23]. Using hepatocellular carcinoma Hep 3B cells, Perng et al. described proapoptotic and anti-inflammatory activities of 2-methoxycinnamaldehyde by inducing the mitochondrial membrane potential loss, cytochrome *c* release, activation of caspase 3 and 9, and DNA content increase in sub G1 phase and downregulation of NF-κB, cyclooxygenase-2 and prostaglandin E2 levels in vitro and in vivo [24]. In addition, anti-inflammatory effects of EOC were observed in a human skin disease model [25]. Finally, EOC was evaluated against human cancer cells of breast adenocarcinoma (MCF7, T47D, and MDA-MB-231), chronic myelogenous erythroleukemia (K562), and neuroblastoma cell lines (SH-SY5Y). Using MTT assay, EOC was very active against all the tested cell lines, while it was more cytotoxic on K562 and less on T47D [26].

Chemopreventive and therapeutic activities of *C. zeylanicum* have not yet been tested in a rodent breast cancer model. The goal of this study was to evaluate the anticancer effects of dietary administered *C. zeylanicum* using chemically induced and 4T1 syngeneic breast adenocarcinoma rat and mouse models. The rationale for this study was based on previous results from our laboratory in which we have documented tumor-suppressive effects of the *Thymus vulgaris* L. haulm, *Syzygium aromaticum* L. buds, *Origanum vulgare* L. haulm, a mixture of fruit peel polyphenols, *Chlorella pyrenoidosa*, and young barley leaves [2,3,4,5,6,11]. These mentioned spices, herbs, fruits, or plant foods showed significant anticancer effects in a rat BC model in vivo and in vitro that were accompanied by significant proapoptotic, antiproliferative, antiangiogenic, antioxidant, epigenetic, or anti-stem cells effects in cancer tissue. As we described previously, a natural cocktail of bioactive molecules included in the mentioned whole plant substances affects a wide signaling network involved in mammary carcinogenesis. For these reasons, our aim in this study was to carry out an extensive analysis of the antitumor activities of *C. zeylanicum* (CIN) using several models of BC. Chemoprevention and allograft models were applied to define cancer risk reduction (tumor frequency) after long-term administration of *C. zeylanicum* or treatment potential (tumor volume) of this spice, respectively. With the aim to analyze the antitumor effects induced by *C. zeylanicum,* the validated markers of apoptosis (caspase-3, Bax, Bcl-2), proliferation (Ki67), angiogenesis (VEGF, VEGFR-2), oxidative damage (MDA), cancer stem cells (CD24, CD44, ALDH1A1, EpCam), and cancer cell epigenetics (methylation status of five gene promoters, four parameters of histone chemical modifications, and expression of six miRNAs) were used. In addition, selected histopathological characteristics of cancer tissue (the ratio of high/low grade carcinomas in rats and mitotic index and tumor necrosis ratio in mice) were assessed. All above-mentioned markers belong amongst the most assessed in oncological practice, and thus we presume that the evaluation of such markers may improve the extrapolation of our data into clinical oncology. The comparison of in vitro and in vivo results may contribute to gaining more valid data. Therefore, we will use two human adenocarcinoma cell lines (MCF-7 and MDA-MB-231) for mechanistic analyses of EOC efficacy (parameters of proliferation, cell cycle, and apoptosis in vitro).

## 2. Results

### 2.1. Plant Secondary Metabolites in C. zeylanicum

**Figure 1:**
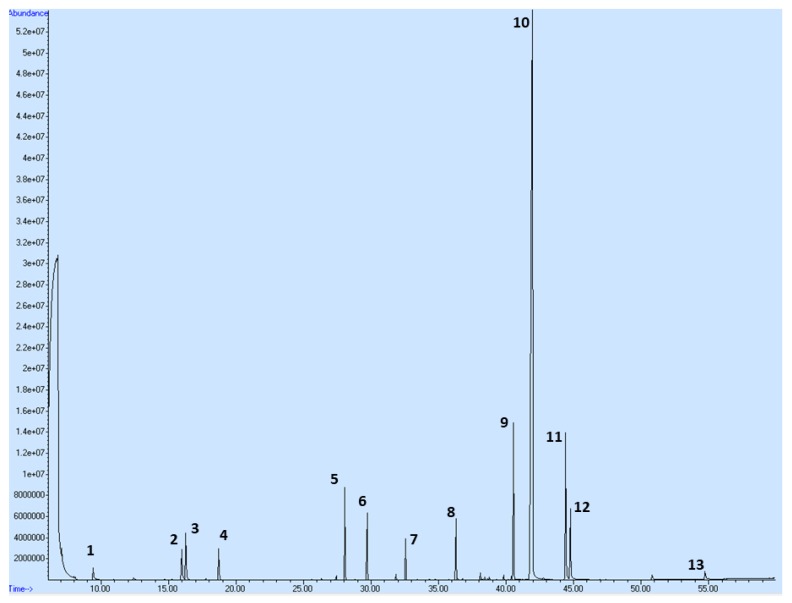
GC-MS chromatogram of the essential oil of *C. zeylanicum* L. bark (EOC) with the most abundant peaks. Numbering of peaks is explained in Table 1.

We performed the phytochemical profiling of the secondary metabolites of the C. *zeylanicum* essential oil used in this pharmacological study based on a Gas Chromatography-Mass Spectrometry (GC-MS) analysis that showed its composition (Table 1 and Figure 1). A total of 47 compounds were observed in the oil; however, only compounds exceeding 0.5% of the total content are shown in Table 1, equating to a total of 97.543%. Cinnamaldehyde, cinnamaldehyde dimethyl acetal, and cinnamyl acetate have been identified as major constituents, representing more than 75% of the sum of compounds in *C. zeylanicum* essential oil.

### 2.2. Parameters of Rat Mammary Carcinogenesis and Histopathology of Tumors

*C. zeylanicum* significantly reduced the tumor incidence in both treated groups by 15.5% (*p* < 0.05) compared to control animals (Table 2). Regarding other parameters of rat mammary carcinogenesis, tumor frequency and latency were not significantly changed in treated groups compared to the control group; however, apparent positive tendencies to decrease tumor frequency and lengthening of tumor latency (both parameters with boundary significance) were found. Higher doses of cinnamon significantly reduced tumor volume by 39% (*p* < 0.05) compared to the group with a lower cinnamon dose (Table 2).

Mixed papillary/cribriform, cribriform/papillary, cribriform, and cribriform/comedic carcinomas were the most common mammary lesions observed in rats. Histopathological analysis of treated rat tumor specimens showed a significant decrease in the ratio of high-/low-grade carcinomas by 61% in CIN 0.1 group (*p* < 0.05) and by 53.5% in CIN 1 group (*p* < 0.05) when compared to controls.

### 2.3. 4T1 Model in Mice

**Figure 2:**
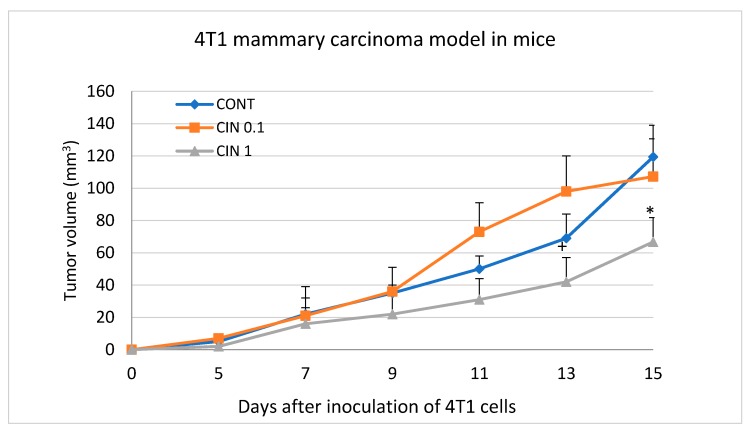
Tumor volume of mouse 4T1 mammary adenocarcinomas during the experiment. Data are expressed as mean ± SEM. Significant differences: * *p* < 0.05 vs. CONT, + *p* < 0.05 vs. CIN 0,1.

**Figure 3:**
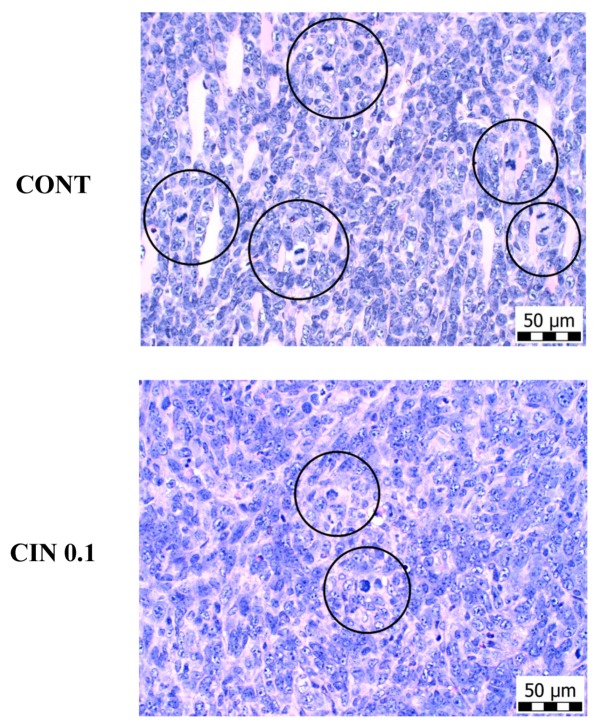

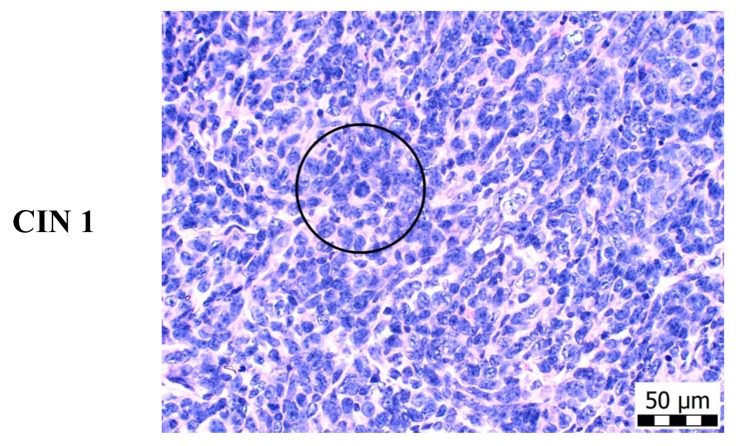
The mitotic activity index after the treatment with *C. zeylanicum* in 4T1 tumors in Balb/c mice. The mitotic figures (prophases, metaphases, anaphases) are highlighted in circles; H&E staining; magnification ×400.

At the end of the experiment, cinnamon in the higher dose significantly reduced the volume of 4T1 tumors by 44% (*p* < 0.05) compared to control mice (Figure 2). Histopathological analysis of 4T1 tumors showed a significant dose-dependent decrease in the mitotic index by 29% (*p* < 0.05) and 45.5% (*p* < 0.001) in treated mice vs. controls (Table 3 and Figure 3). Evaluating the ratio of necrosis/whole tumor area, we did not find any significant changes between control and treated groups.

### 2.4. Immunohistochemistry of Rat Tumors

**Figure 4:**
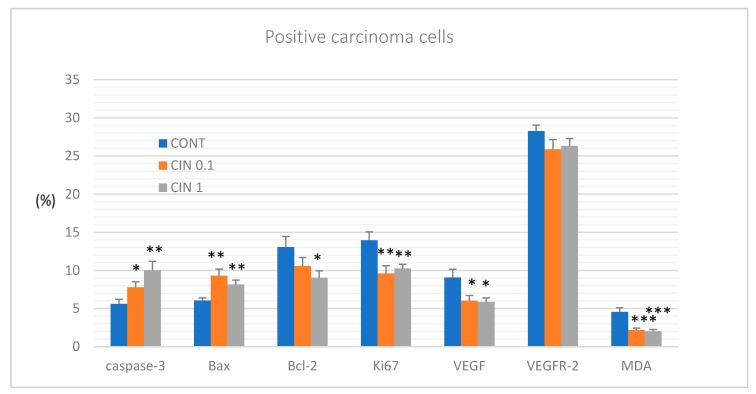
Immunohistochemical evaluation of caspase-3 (cytoplasmic), Bax, Bcl-2, Ki67, VEGFA, VEGFR-2, and MDA expression in rat mammary carcinoma cells after the administration of *C. zeylanicum* in two doses. Data are expressed as mean ± SEM. Significant difference, * *p* < 0.05, ** *p* < 0.01, *** *p* < 0.001 vs. CONT. Figures represent the expression of proteins quantified as the average percentage of antigen positive area in standard fields (0.5655 mm^2^) of tumor hotspot areas. The values of protein expression were compared between treated (CIN 0.1, CIN 1) and non-treated (control) carcinoma cells of female rats; > 60 images for one marker were assessed.

Figure 4 summarizes the analysis of apoptotic (cytoplasmic caspase-3 expression, Bax and Bcl-2), proliferative (Ki67 expression), and angiogenic markers (VEGF and VEGFR-2 expression), as well as the antioxidant effect (MDA levels) in rat mammary carcinoma cells in vivo. Cinnamon at a lower dose increased caspase-3 expression by 38.5% (*p* < 0.05), Bax expression by 53.5% (*p* < 0.01), but decreased Ki67 expression by 31.5% (*p* < 0.01), VEGF expression by 33.5% (*p* < 0.05) and MDA levels by 35.5% (*p* < 0.05) compared to the control group. A higher cinnamon dose increased caspase-3 expression by 78.5% (*p* < 0.01), Bax expression by 35% (*p* < 0.01), and decreased Bcl-2 expression by 30.5% (*p* < 0.05), Ki67 expression by 26.5% (*p* < 0.01), VEGF expression by 35% (*p* < 0.05), and MDA levels by 55.5% (*p* < 0.001) in comparison with controls. VEGFR-2 expression was not significantly altered in treated cancer cells compared to control cells.

**Figure 5:**
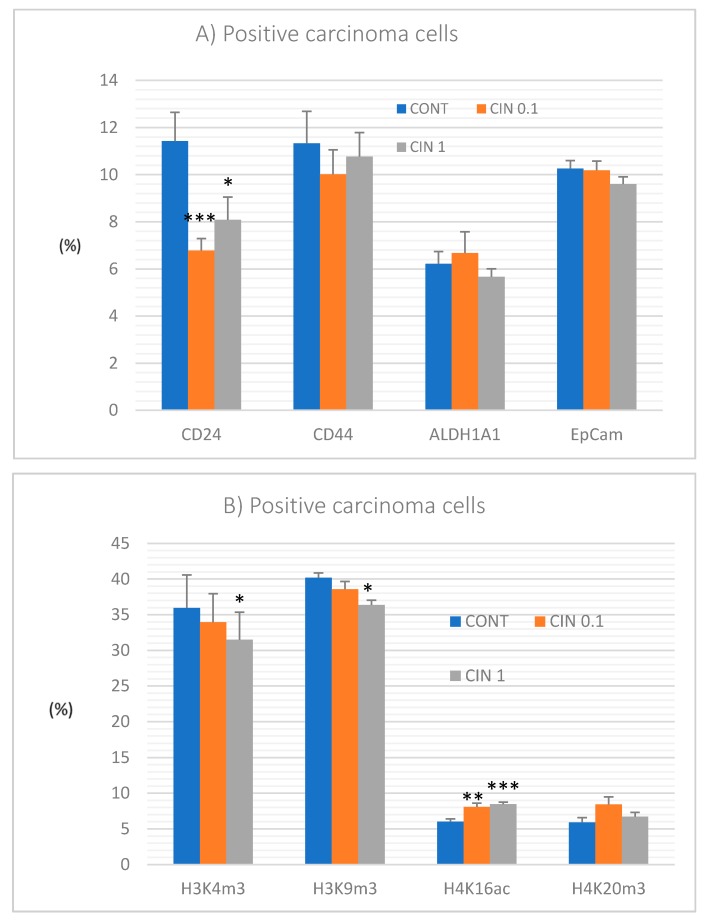
Immunoexpression of cancer stem cell (**A**) and epigenome (**B**) markers in rat mammary carcinoma cells after treatment with *C. zeylanicum*. Data are expressed as mean ± SEM. Significant difference: * *p* < 0.05, ** *p* < 0.01, *** *p* < 0.001 versus CONT. The values of protein expression were compared between treated (CIN 0.1, CIN 1) and non-treated (control) carcinoma cells of female rats; at least 60 images for one marker were analyzed.

Evaluation of cancer stem cells (CSCs) parameters showed a dose-independent significant decrease in CD24 expression by 40.5% (*p* < 0.001) and 29% (*p* < 0.05) in treated groups compared to the control group. Other CSCs parameters such as CD44, ALDH1A1, and EpCam did not significantly differ in the treatment groups vs. controls (Figure 5A).

Post-translation histone 3 and histone 4 chemical modifications induced by cinnamon in higher doses demonstrated significant decreases in H3K4m3 by 12.5% (*p* < 0.05) and H3K9m3 by 9.5% (*p* < 0.05) when compared to controls. Levels of H4K16ac in treated cancer cells were dose dependently increased by 34% (*p* < 0, 01) and 40.5% (*p* < 0, 001) in comparison with controls. Changes in H4K20m3 levels in treated groups vs. the untreated group were not significant (Figure 5B).

**Figure 6:**
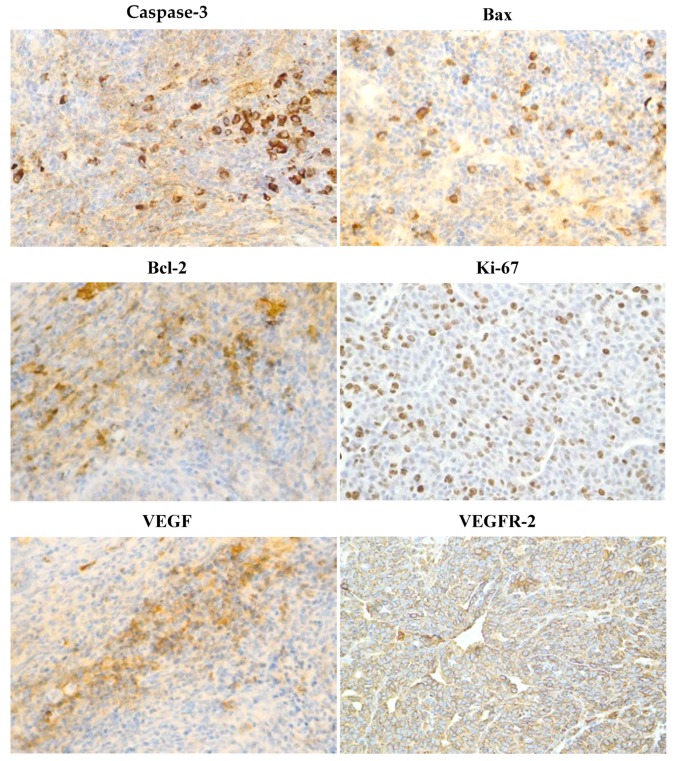

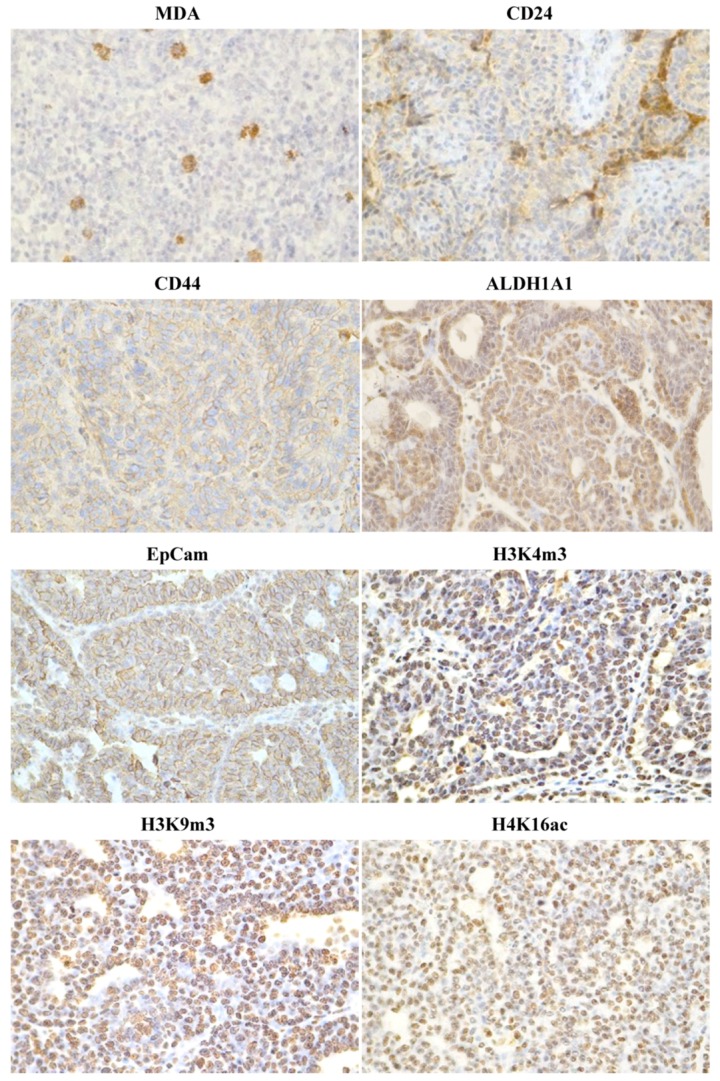

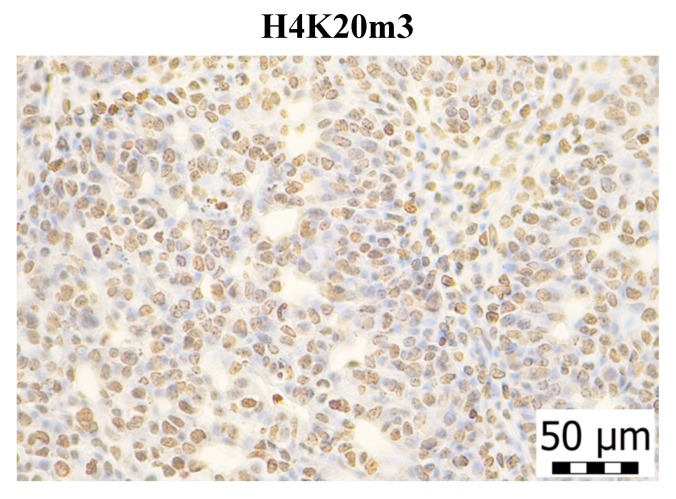
Representative images of expression of caspase-3, Bax, Bcl-2, Ki67, VEGFA, VEGFR-2, MDA, CD24, CD44, ALDH1A1, EpCam, H3K4m3, H3K9m3, H4K20m3, and H4K16ac in rat carcinoma tissue of mammary gland. For detection, polyclonal caspase-3 antibody (Bioss, Woburn, MA, USA), polyclonal Bax and Bcl-2 antibodies (Santa Cruz Biotechnology, Paso Robles, CA, USA), monoclonal Ki67 antibody (Dako, Glostrup, Denmark), monoclonal VEGFA and VEGFR-2 antibodies (Santa Cruz Biotechnology, Paso Robles, CA, USA), polyclonal CD24 antibody (GeneTex, Irvine, CA, USA), polyclonal CD44 antibody (Boster, Pleasanton, CA, USA), polyclonal ALDH1A1 antibody (ThermoFisher, Rockford, IL, USA), polyclonal MDA, EpCAM, H3K4m, H3K9m3, and H4K20m3 antibodies (Abcam, Cambridge, MA, USA) and monoclonal H4K16ac antibody (Abcam, Cambridge, MA, USA) were applied; final magnification: ×400.

Representative pictures of the expressions of caspase-3, Bax, Bcl-2, Ki67, VEGFA, VEGFR-2, MDA, CD24, CD44, ALDH1A1, EpCam, H3K4m3, H3K9m3, H4K16ac, and H4K20m3 in rat mammary carcinomas are shown in Figure 6.

### 2.5. miRNA Expression

**Figure 7:**
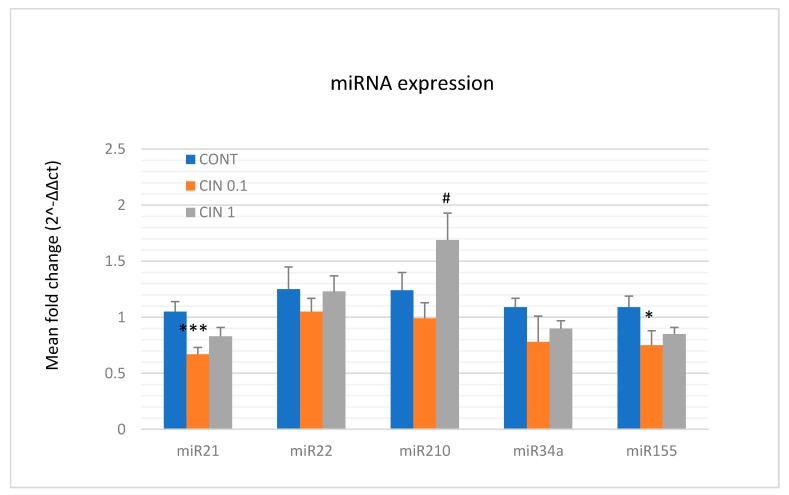
Relative miRNA expression of miR21, miR22, miR210, miR34a, and miR155 in rat mammary carcinomas. MiR-191-5p was selected as the internal control miRNA to normalize the cDNA levels of the samples. Data are expressed as mean ± SEM. Significant difference, * *p* < 0.05, *** *p* < 0.001 vs. CONT, ^#^ *p* < 0.05 vs. CIN 0.1.

With the aim to perform more precise analysis of the oncostatic and epigenetic potential of *C. zeylanicum,* we also evaluated the expression of several well-validated miRNAs in rat mammary cancer tissue in vivo (Figure 7). *C. zeylanicum* in a lower dose significantly decreased the expression of oncogenic miRNAs - miR21 by 36% (*p* < 0.001) and miR155 by 31% (*p* < 0.05) when compared with the control. In the higher dose, we found an up-regulation of tumor-suppressive mi210 expression by 70.5% (*p* < 0.05) when compared to the lower dose. *C. zeylanicum* did not change the expressions of tumor-suppressive miR22 and miR34a compared to the control group.

### 2.6. Quantitative Methylation Analysis

**Figure 8:**
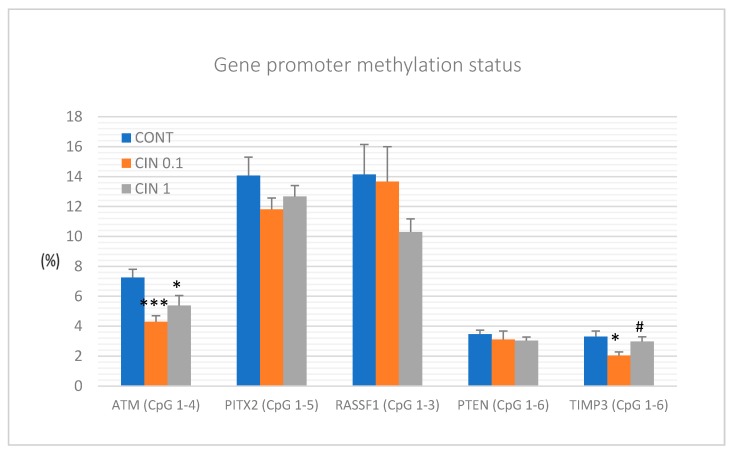
Total DNA gene promoter methylation status of ATM, PITX2, RASSF1A, PTEN, and TIMP3 genes in rat mammary carcinomas. Total promoter methylation status was calculated from all evaluated CpG isles of ATM, PITX2, RASSF1A, PTEN, and TIMP3 in carcinoma tissue from control and treated groups. Significant difference, * *p* < 0.05, *** *p* < 0.001 vs. control and # *p* < 0.05 vs. CIN 0.1.

The methylation status of five tumor-suppressor gene promoters was analyzed: *ATM* including four evaluated CpG sites (CpG 1-4), *PITX2* (CpG 1-5), *RASSF1* (CpG 1-3), *PTEN* (CpG 1-6), and *TIMP3* (CpG 1-6) (Figure 8). We evaluated twenty rat tumor samples for each experimental group (CONT, CIN 0.1, and CIN 1). *C. zeylanicum* significantly and dose-independently reduced the total methylation status of ATM gene promoter by 40.5% (*p* < 0.001) and 26% (*p* < 0.05) vs. control group. Cinnamon in a lower dose significantly decreased methylation in the TIMP3 gene promoter by 38.5% (*p* < 0.01) when compared to the control group; however, a higher dose of cinnamon was less effective (decrease by 10% vs. control, *p* > 0.05). The comparison between control and treated groups did not show any significant differences in total promoter methylation of *PITX2*, *RASSF1*, and *PTEN* genes; however, in all three cases, apparent tendencies in the decrease of specific parameters were found (Figure 8).

### 2.7. Physiological in Vivo Effects

Compared to control group, a higher cinnamon dose increased serum HDL-cholesterol level by 14% (*p* < 0.05) and a lower cinnamon dose decreased serum glucose level by 18.5% (*p* < 0.001). Cinnamon administered in lower doses showed hyperlipidemic effects characterized by an increase in serum triacylglycerols by 30.5% (*p* < 0.05) and VLDL-cholesterol by 31.5% (*p* < 0.05) vs. controls (data not shown). At the end of the experiment, we did not observe any changes in body weight in either rats or mice. However, there was a significant increase in the food intake of rats by 2.0 g (CIN 0.1) and 4.4 g (CIN 1) when compared to the control group (15.0 g of diet/rat/day). Chronic administration of cinnamon during 14 weeks in rats was well tolerated without macroscopic changes in organs (evaluation of liver steatosis, hepato/splenomegaly, gastritis), hematopoietic disorders, and other side effects (vitality, hair, mucosa). The average daily dose of cinnamon per rat was 16.99 mg in the CIN 0.1 group and 193.7 mg in CIN 1 group. The daily average doses of cinnamon per mouse were 7.1 mg (CIN 0.1) and 72 mg (CIN 1).

### 2.8. In Vitro Analyses on MCF-7 and MDA-MB-231 Cells

**Figure 9:**
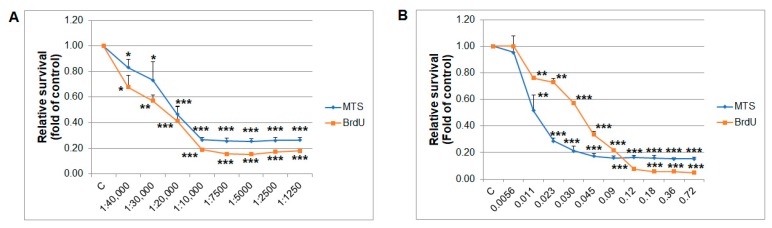
Relative survival of MCF-7 (**A**) and MDA-MB-231 (**B**) cells treated with EOC (1:1250-1:40,000/160,000) and analyzed by MTS and BrdU incorporation assays. Data were obtained from three independent experiments and significant differences were marked as *p* < 0.05 (*), *p* < 0.01 (**), *p* < 0.001 (***) vs. control cells (untreated).

MTS and BrdU incorporation assays were used to evaluate the antiproliferative effects of EOC on MCF-7 and MDA-MB-231 cells. Results showed that EOC significantly decreased metabolic activity followed by decreased cell survival in a dose- and time-dependent manner in both cell lines (Figure 9). Comparing cell lines, MDA-MB-231 cells showed significantly higher sensitivity to EOC treatment compared to MCF-7 cells. Based on the mentioned analyses, we used different final EOC dilutions for MCF-7 and MDA-MB-231 cell lines in follow-up in vitro evaluations (1:25000 or 1:65000, respectively).

**Figure 10:**
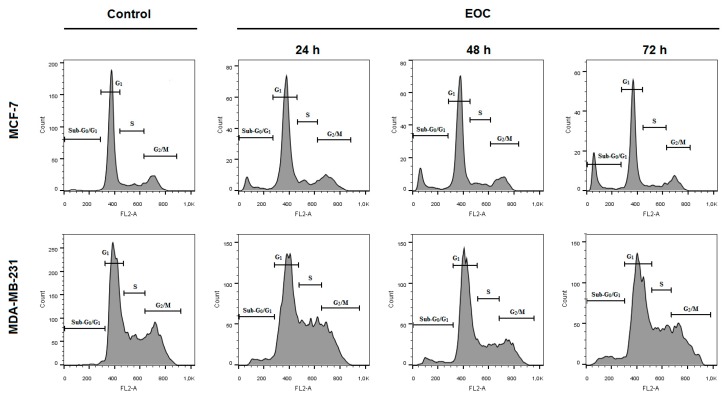
Representative diagrams of cell cycle distribution in MCF-7 and MDA-MB-231 cells after EOC treatment (1:25,000, 1:65,000).

The flow cytometric analyses in MCF-7 and MDA-MB-231 cells after EOC treatment were assessed after 24, 48, and 72 h. The evaluation of cell cycle progression (Table 4 and Table 5; Figure 10) after EOC treatment in MCF-7 and MDA-MB-231 cells showed significant differences between both cell lines. In MCF-7 cells, we noticed increased accumulation of cells in sub-G_0_/G_1_ population (recognized as apoptotic with fractionated DNA) after 24 h to 72 h of EOC treatment with concomitant decrease of cells in G1 phase of cell cycle. On the other hand, cell cycle analyses of MDA-MB-231 cells showed a delayed increase of cells in sub-G_0_/G_1_ population with concomitant decrease in G1 phase only after 72 h. Moreover, S phase cell accumulation occurred after 72 h of EOC-treatment.

**Figure 11:**
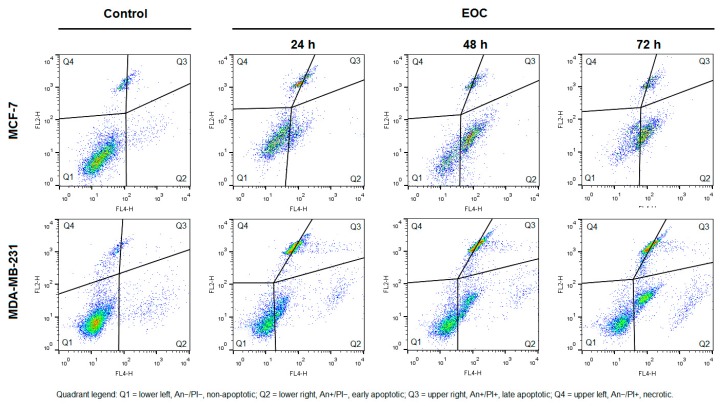
Representative diagrams of apoptotic cell diversification in MCF-7 and MDA-MB-231 cells after EOC treatment (1:25,000, 1:65,000).

An increased population of MCF-7/MDA-MB-231 cells in sub-G_0_/G_1_ fraction suggested an induction of apoptosis after EOC treatment. Analysis of annexin V positivity, a marker of programmed cell death induction, showed significant phosphatidyl serine (PS) externalization after 24, 48, and 72 h of EOC treatment in both cell lines (Table 6 and Table 7; Figure 11). Annexin V assay also showed different diversification of cell population between cell lines. In MCF-7 cells, EOC treatment (24–72 h) significantly increased in cells in the early stage of apoptosis (68.60% on 72 h) with a lack of cells in late apoptotic/necrotic phase after 48 h (about 5%–8%). On the other hand, EOC treatment (24–72 h) in MDA-MB-231 cells significantly increased cells in the early/late apoptotic stage in a time-dependent manner with a subsequent increase in cell death. In summary, MDA-MB-231 cells showed only 54% of annexin V positivity after 72 h of EOC treatment compared to 74% positivity in MCF-7 cells.

**Figure 12:**
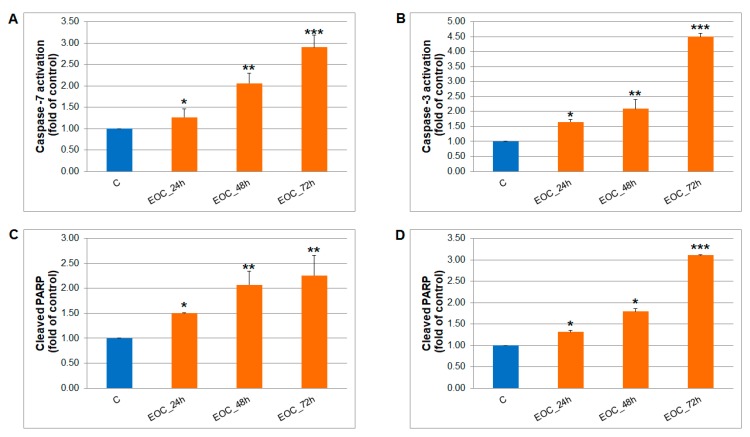
Effect of EOC treatment (1:25,000, 1:65,000) on: **A**) caspase-7 (MCF-7), **B**) caspase-3 activation (MDA-MB-231) and PARP cleavage in both cell lines (**C**, MCF-7 cells; **D**, MDA-MB-231 cells) analyzed by flow cytometry. Data were obtained from three independent experiments and significant differences were marked as *p* < 0.05 (*), *p* < 0.01 (**), *p* < 0.001 (***) vs. control cells (untreated).

**Figure 13:**
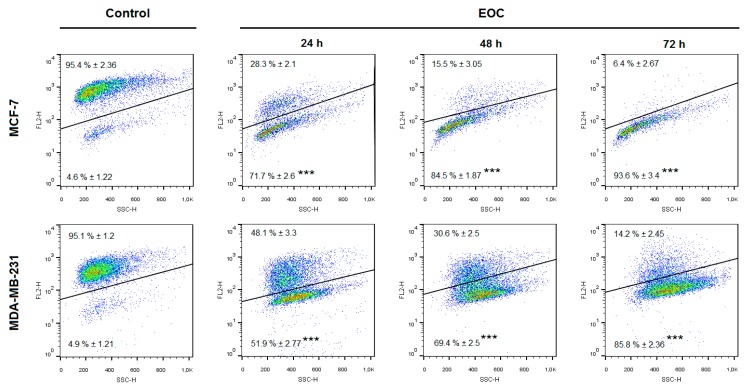
Effect of EOC treatment on mitochondrial membrane potential (MMP) changes in MCF-7 and MDA-MB-231 cells. Data were obtained from three independent experiments and significant differences were marked as *p* < 0.001 (***) vs. control cells (untreated).

**Figure 14:**
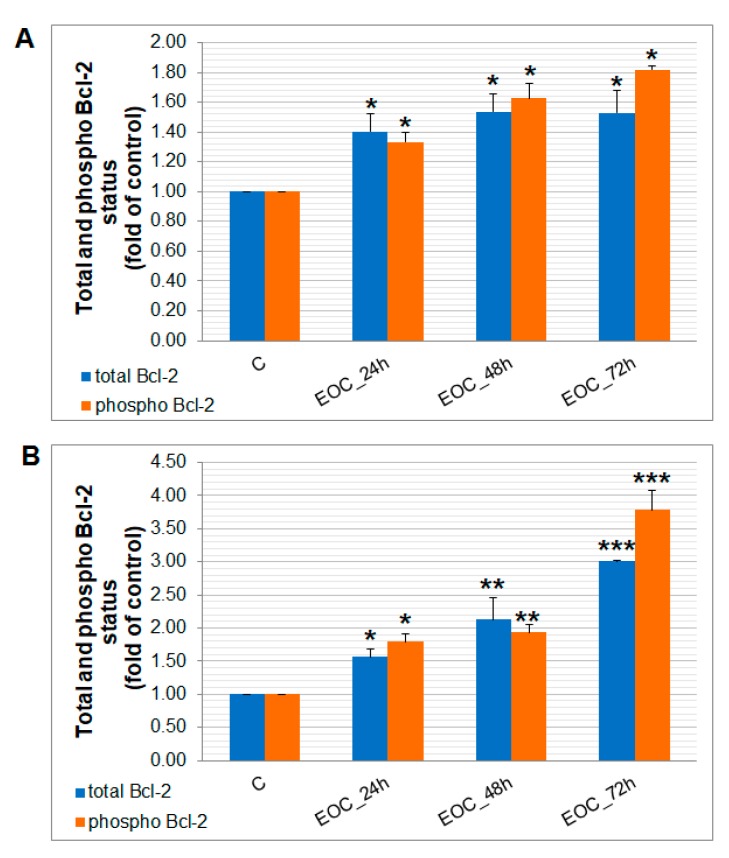
Distribution and activity of antiapoptotic mitochondria-associated protein Bcl-2 after EOC treatment (1:25,000, 1:65,000) in MCF-7 (**A**) and MDA-MB-231(**B**) cells analyzed by flow cytometry. Results are expressed as mean values of three independent experiments and significant differences were marked as *p* < 0.05 (*), *p* < 0.01 (**), *p* < 0.001 (***) vs. untreated control.

The caspase-dependent pathway of cell death in MCF-7 and MDA-MB-231 cells was confirmed after analyses of caspase-7 or caspase-3 activation (Figure 12). Both cell lines showed similar time-dependent trends of caspase activation after EOC treatment leading to similar Poly (ADP-ribose) polymerase (PARP) cleavage rate. Furthermore, time-dependent depletion of mitochondrial membrane potential (MMP) in both MCF-7 and MDA-MB-231 cells occurred after EOC treatment (Figure 13). Due to mitochondrial stress after EOC treatment, a significant release (increase) of antiapoptotic Bcl-2 (Figure 14) protein complexes from mitochondria to cytosol occurred quickly, after 24 h. Analysis of phosphorylation status clearly showed pro-apoptotic deactivation of Bcl-2 (Figure 14) and clearly demonstrated activation of the mitochondrial apoptosis pathway in the same time. The MDA-MB-231 cell line showed significantly stronger Bcl-2 release and deactivation after EOC treatment compared to MCF-7 cells.

## 3. Discussion

As some phase II clinical trials showed [27,28,29], the anticancer effects of selected phytochemicals or whole plant foods characterized by a low toxicity in humans during long-term administration may provide a progressive clinical approach to cancer patients or high-risk individuals. With the aim to uncover new prospective plant substances with prominent anticancer efficacy that can be potentially introduced within novel therapeutic/preventive clinical utilities, we aimed to evaluate the oncostatic effects of *C. zeylanicum* in BC animal models and human cell lines.

Generally, the effective dosage of therapeutics including whole plant foods is very specific for each mammalian species. Therefore, only well-controlled clinical studies can provide information on the appropriate dosage regimen in high-risk individuals or patients. The doses of cinnamon used in this study were derived from our previous experiences with the chemically-induced and allograft mammary carcinoma rodent models [2,3,4,5,6,7]. Our results from this and all our previous studies demonstrated the relevance of the used dosage. In the chemopreventive study in rats, both higher and lower doses of cinnamon significantly reduced the incidence of mammary tumors and non-significantly reduced their frequency by more than 30% compared to controls. In the same study, cinnamon significantly improved histopathological characteristics of mammary carcinoma lesions (HG/LG ratio) in both treated groups. Cinnamon did not show chemopreventive efficacy in rats, but did show a significant therapeutic effect in the 4T1 model of breast adenocarcinoma in mice. The dominant substances present in tested cinnamon are cinnamaldehyde, cinnamaldehyde dimethyl acetal, cinnamyl acetate, eugenol, linalool, or eucalyptol (Table 1) that have been already been proven as molecules with significant anticancer activities against different cancer cell lines, including BC [21,30,31,32,33,34,35]. In our previous study, T. vulgaris L., applied in the same mouse model and doses as cinnamon, demonstrated significant reduction of 4T1 tumor volume in mice by more than 80% compared to the control group [7]. Mentioned significant therapeutic effects of plant substances are comparable with the antitumor efficacy of several synthetic drugs tested in the 4T1 mouse model of breast cancer [36,37,38]. The results of this and our previous preclinical rodent model studies confirmed our scientific assumption that the significant anticancer effect of whole plant functional foods is based more on a combination of several phytochemicals than on isolated molecules [2,3,4,5,6,7,39,40]. Numerous papers describe that dominant metabolites found in our EOC (cinnamaldehyde, eugenol, linalool, eucalyptol, limonene) are absorbed via rat GIT and are consequently significantly effective in the organism [41,42,43,44,45]. There is no reason to doubt that dominant metabolites from EOC were present in rat plasma after eating cinnamon in this study. We strongly assume that targeting multiple signaling pathways in cancer cells by many bioactive molecules present in *C. zeylanicum* at the same time may represent a more effective biomedical approach in oncology. Recently, our group published a comprehensive and critical review comparing application of natural phyto-complexes vs. isolated compounds using different cancer models. In this review paper, we clearly summarized significantly higher cancer risk reduction activity in natural phyto-complexes when compared to isolated molecules [1]. Finally, significant (in many cases robust) chemopreventive effectiveness of various plant whole foods (dark fruits, herbs) in mammary carcinogenesis in female rats has also been described by other authors [46,47,48,49]. The translation of data from animal studies (such as daily doses of plant foods suitable for humans or effective plasma levels of plant metabolites) to clinical practice is not easily applicable. Different mammal species show different pharmacodynamics/pharmacokinetics of phytochemicals; therefore, effective plasma levels of plant metabolites must be derived strictly from clinical research.

We suppose that the antitumor effects of the natural plant substances tested by our group are based on the proapoptotic, antiproliferative, antiangiogenic, or antioxidant mode of action of a specific cocktail of phytochemicals. Programmed cell death can be activated by an internal or external apoptotic pathway. We evaluated the former, i.e., mitochondria-induced apoptosis in vivo. Protein Bcl-2 plays a key role in the regulation of apoptosis in which an increasing Bax/Bcl-2 ratio enhances caspase-3 activity and subsequently induces apoptosis in tumor cells [50]. Phytochemicals seem to have an important regulatory role in Bax/Bcl-2/caspase-3 signaling in breast carcinogenesis [51,52]. In this study, cinnamon caused a significant increase in the Bax/Bcl-2 ratio, which correlated with an increase in caspase-3 expression in treated mammary tumors in rats. In our previous chemopreventive studies in female rats, we also demonstrated a significant correlation between an increase in Bax/Bcl-2 ratio and an increase in caspase-3 expression in mammary cells in vivo after the application of dark fruit peel, oregano, and clove buds [3,5,6]. The pro-apoptotic effects of cinnamon were also evaluated in our parallel in vitro study. Firstly, we observed a decrease in cell viability of the MCF-7 cell line using EOC. These changes were associated with an increase in the population of MCF-7 cells in the sub-G0/G1 phase. In MDA-MB-231 cells, we found a delayed increase of cells in G0/G1 phase and an increase in S phase of the cell cycle. These data indicate the blockage of cell cycle progression and induction of apoptosis in MCF-7 and MBA-MD-231 cells after the treatment with EOC. Furthermore, we detected early and late stages of apoptosis or necrosis in the MDA-MB-231 cell lines and early apoptotic stage in MCF-7 cells using Annexin V/PI staining. Moreover, we observed an increase in the mitochondrial membrane potential induced by EOC. It is known that such changes modulate the activity of key apoptotic regulators [53]. In this regard, our in vitro study demonstrated antiapoptotic inactivation of Bcl-2. The decreased expression of Bcl-2 protein induces the release of cytochrome *c* from mitochondria to the cytoplasm and ultimately results in apoptosis via an activation of caspases [54,55]. In the relevant cell line, we also demonstrated an activation of caspase-7 or caspase-3 after EOC application leading to an increase in PARP cleavage rate. In accordance with results of this in vitro study, we noted significant proapoptotic effects of the essential oils of thyme, clove, and oregano and extracts of young barley leaves, fruit peel polyphenols, and *Ch. Pyrenoidosa* [2,3,4,5,6,7] in our recent in vitro experiments (using of MCF-7 or MDA-MB-231 cells). Based on these data, the above-mentioned plant substances represent potentially effective apoptosis-inducing foods in human BC.

Numerous preclinical studies have described the antiproliferative and antiangiogenic potential of phytochemicals in mammary carcinogenesis [56,57,58,59,60]. Many phytochemicals were documented as molecules that directly target estrogen-dependent and estrogen-independent signaling associated with the proliferation of the mammary gland cells. This signaling includes several important mechanisms/pathways such as COX-2, Hedgehog, Nrf2, NF-κB, poly-ADP-ribosylation, Wnt, PI3 kinase, Plk1, STAT3, or epigenetic modifications [61]. The Ki67 protein is a prominent marker associated with cell proliferation. The immunohistochemical analysis of this protein in our study demonstrated a significant decrease in rat mammary carcinoma cells in vivo after treatment with both doses of cinnamon when compared to controls. Moreover, histopathological evaluations revealed a significant decrease in mitotic index in mouse 4T1 cells in vivo after long-term administration of cinnamon. These results are in an accordance with our earlier studies in which we tested plant functional foods (young barley, dark fruit peels, oregano, and clove buds) that showed significant antiproliferative activities in rat and mouse mammary carcinoma models [3,4,5,6]. Using MTT and BrdU proliferation assays, we confirmed our results obtained from animal model studies. The cinnamon essential oil reduced the viability and metabolic activity of MCF-7 and MDA-MB-231 cell lines. The antiproliferative effect of cinnamon in human breast adenocarcinoma cells was also confirmed by blocking cell cycle progression. Despite the above-mentioned optimistic results of our parallel in vivo and in vitro studies, it is not always easy to find the same accordance in results as was shown in our previous experiments with *Ch. pyrenoidosa* and *T. vulgaris* L. haulm [2,7]. Based on these results, we can conclude that the antiproliferative activity of phytochemicals present in various plant functional foods depends on used cancer models/cell lines that are characterized by various genotypes and phenotypes.

Investigating the therapeutic possibilities of various molecules affecting signaling pathways of angiogenesis (e.g., VEGF, EGF, FGF, HGF, tyrosine kinases receptors, neuropilins, integrins) is an intensively developing area of oncology research. The disruption of the VEGF-kinase ligand/VEGF receptor signaling pathway and regulation of other molecules that interact with VEGF signaling may play an important role in the suppression of angiogenesis and vasculogenesis in cancer tissue [62]. Natural plant products rich in bioactive phytochemicals that interact with VEGF-promoting factors and consequently suppress angiogenic signaling pathways have been documented to inhibit cancer growth [63,64,65,66]. We found that both doses of cinnamon significantly reduced VEGF expression in mammary rat carcinoma in vivo. In our previous study, we demonstrated a significant decrease in VEGFR-2 expression in a dose-dependent manner after thyme treatment in rat tumors in vivo [7]. Furthermore, other recent animal studies in our laboratory showed that natural mixtures of phytochemicals present in whole plant foods have a great potential in suppressing the angiogenesis in mammary tumor tissue [4,5,6]. However, in the case of plant substances tested by our group, further studies must be specifically directed to the individual mechanisms of angiogenesis modulation in cancer tissue, thus defining their exact roles in the promotion and progression phases of carcinogenesis.

The imbalance in the redox status of the cell leads to an oxidative stress and consequently (via the high reactivity of free radicals) to oxidative damage of principal cell molecules (such as DNA, proteins, lipids, or oligo/polysaccharides). Mentioned changes in the cell play an essential role in the etiopathogenesis of neoplasia [67,68]. We have shown that a mixture of natural phytochemicals contained in whole plant functional foods such as cinnamon, young barley, clove buds, and thyme significantly reduced oxidative damage of cellular lipids or proteins [4,5,7]. Malondialdehyde (MDA) is the most commonly used marker of oxidative damage of lipids by free radicals [69]. Our data pointed to a significant decrease of MDA levels in rat tumors, thus demonstrating the antioxidant activity of *C. zeylanicum* L., *S. aromaticum* L., *T. vulgaris* L. and pointing to their genoprotective effects. Using the same model, young barley leaves demonstrated the reduction of levels of dityrozines in cancer cells [4]. Research by other authors that described rather pro-oxidative effects of polyphenols, terpenoids, and other plant-derived antioxidants is mostly limited by the use of very high doses and an in vitro experimental approach [70]. These experimental conditions thus cannot consistently mimic the situation of in vivo systems, including the human organism.

Preclinical studies clearly indicated significant anticancer effects of phytochemicals mediated by CSCs targeting via the modulation of signaling pathways such as Wnt, Notch, Hedgehog, or others, as well as via regulation of mechanisms involved in the processes of apoptosis or drug resistance [71,72,73]. In clinical practice, there are several established markers of breast CSCs. The phenotype of CD44^+^/CD24^-/low^ and overexpression of ALDH1, EpCAM, and nestin suggest a worsened prognosis of BC in women [74]. CD24, CD44, EpCAM, and ALDH1 have been shown to be good markers of CSCs also in chemically induced mammary carcinogenesis in rats [75,76]. Based on comparative preclinical oncology studies, plant functional foods (containing mixtures of phytochemicals) are supposed to exhibit better anticancer activities (including the anti-CSC properties) when compared to isolated phytochemicals [1]. This study revealed significant reduction of the expression of CD24 in rat tumors after the treatment with both doses of cinnamon. The results of our previous experiment with clove buds showed a significant decrease in CD24 and CD44 expression, and on the other hand, an increase in ALDH1A1 expression in treated mammary carcinomas [5]. Similarly, a study with thyme haulm showed a significant decrease in CD44 and ALDH1A1 expression in tumor tissues in rats [7]. Our testing of oregano using the same model of chemically induced rat mammary carcinogenesis demonstrated decrease in CD24 and EpCAM expression in tumor cells [6]. The data from our laboratory clearly showed the positive effect of plant foods/phytochemicals on clinically relevant parameters of breast CSCs. Clinical research assessing the effects of phytochemicals on CSCs is apparently lagging behind preclinical evaluations and only a few trials could be found. In this regard, a large number of clinical studies focus on mechanisms on how synthetic drugs affect CSCs vitality; however, evidence of plant-derived foods/isolated molecules as anti-CSC agents is lacking. Our results and data from other authors point to significant anti-CSCs effects of phytochemicals in a wide range of cancer types. These activities of phytochemicals are proposed to be mediated via influencing the multiple cell signalings that demonstrate an urgent need for their in-depth investigation within clinical research [77,78,79].

It is well described that epigenetic alterations dynamically contribute to cancer pathogenesis [80]. Numerous phytochemicals with antitumor properties have also shown a significant effect on epigenome of neoplastic cells [10,11,12]. Therefore, the role of a diet rich in phytochemicals and its impact on the cancer epigenome is a clinically highly relevant topic. In this regard, current oncology research is aimed at defining specific epigenetic modulations induced by plant bioactive molecules. They include a global methylation status of oncogenes and tumor-suppressor genes, histone chemical modifications, and non-coding RNA-associated multi-gene control [80]. Therefore, it seems logical that the combination of several isolated phytochemicals or the use of natural cocktails of phytochemicals present in plant foods could provide additive or synergistic effects on numerous epigenetic targets compared to the use of one molecule. Preclinical in vivo studies focusing on a mixture of phytochemicals in naturally occurring plants and their impact on the regulation of promoter methylation patterns and post-translational histone modifications (PHMs) in various malignancies demonstrated optimistic results. All epigenetic markers chosen for our study represent well-described and validated cancer diagnostic and prognostic measures that are applied in both clinical approaches and cancer rodent models. Decreased expression of tumor-suppressor genes such as *ATM* serin/threonine kinase, paired-like homeodomain transcription factor, Ras-association domain family 1, isoform A, Phosphatase and tensin homolog, and Tissue inhibitor of metalloproteinase-3 is frequently observed in patients with BC [81] or in cancer models [7]. By evaluation of the methylation status of selected CpG islands in the promoter regions of the above-mentioned tumor suppressors, we revealed significant decreases in the methylation of *ATM* and *TIMP3* gene promoters in rat mammary cancer tissue in vivo after cinnamon treatment. Regarding TIMP3 gene promoter, the effect was observed with the lowest dose of cinnamon (0.1%) and not with the highest one. It is very difficult to say what the reason for this effect is. It seems likely that some biological effects in vivo need specific concentrations of biologically active molecules. In the case of TIMP3 promoter methylation, the lower concentration of cinnamon triggered mechanisms for downregulation of DNMTs activities; however, our data showed that the higher concentration of cinnamon blocked these mechanisms. In another study, our group demonstrated that the mixture of phytochemicals present in *T. vulgaris* L. caused significant decreases in the methylation patterns of *ATM, RASSF1, PTEN,* and *TIMP3* gene promoters in the mentioned rat model [7]. Finally, our experiment focusing on epigenetic modulation of clove buds showed a demethylation of the promoter of *RASSF1* tumor suppressor in vivo [5]. In studies of other authors, dietary application of black raspberries (BRB) rich in numerous flavonoids such as anthocyanidins led to demethylation of tumor-suppressor gene promoters, including *WIF1*, *SOX17,* and *GKI* in precancerous colon cancer in vivo [82]. Another study testing BRB showed a demethylation effect via reduction of the methylation status of the *Sfr4* promoter region in a rat esophageal squamous cell papilloma model [83].

Regarding post-translational chemical modifications of histone molecules, cinnamon in this study decreased H3K4m3 and H3K9m3 levels and increased H4K16ac levels in rat mammary carcinomas. Moreover, we found that clove buds significantly increased H4K20me3 and H4K16ac levels [5] and *T. vulgaris* decreased H3K4me3 levels in chemically induced rat mammary carcinogenesis [7]. Importantly, all these changes represent a positive impact on epigenetic modifications described by our group in a rat model of BC. Other authors showed that resveratrol restrained a suppressive state of critical tumor-suppressors including BRCA1, p53, and p21 in MCF-7 and MDA-MB-231 cell lines, which led to the inhibition of cancer growth. The cancer inhibition was linked to the decrease in H4R3me2s and H3K27me3 and increase in H3K9ac and H3K27ac levels of histones surrounding promoters of these genes [84]. In another study, the combination of sulphoraphane and Withaferin A downregulated histone deacetylase (HDAC) expression at multiple levels in MCF-7 and MDA-MB-231 cell lines. Authors concluded that lowered levels of HDAC were associated with the decrease in cell viability and induction of apoptosis in both cell lines [85]. The same combination of phytochemicals increased histone methylation levels and thus inhibited cell cycle progression in MCF-7 and MDA-MB-231 [57]. It is already well documented that phytochemicals or whole plant foods sensitize tumor cells through different epigenetic targets including oncogenes and tumor-suppressor genes as well as DNA methyltransferases (DNMTs) and other epigenetic mechanisms such as chemical modifications of histone molecules [86]. Deeper understanding of the promoter methylation patterns and the global patterns of PHMs and their consequences for chemical modifications of nuclear chromatin may reveal important molecular targets for dietary phytochemicals that can be clinically applied as progressive tools towards cancer disease.

MiRNAs, small non-coding RNAs, function as post-transcriptional regulators of gene expression. Accordingly, miRNAs have been documented to regulate a plethora of target genes that are involved in the processes of carcinogenesis. Numerous (mainly preclinical) studies described that phytochemicals effectively target miRNAs which represents another mechanism of their anticancer action [13,87,88,89]. A number of miRNAs are evidenced as valid diagnostic and prognostic markers of BC. MiR21 and miR155 represent oncogenic miRNAs, while miR22 and miR34a are well documented as tumor-suppressive miRNAs within clinical and preclinical BC research [90,91,92,93]. MiR210 was described as oncogenic in hypoxic conditions, and on the other hand as a tumor suppressor in normoxic cells e.g., during the initiation phase of oncogenesis as was described in the chemoprevention in vivo BC model [7,94]. In this study, the lower dose of cinnamon significantly decreased expressions of miR21 and miR155, and the higher dose showed the same tendencies. Evaluating remaining miRNAs in this study, we did not find any significant changes or contradictory results when compared to data of other authors. Recently we have documented very consistent in vivo data when *T. vulgaris* caused significant increase in miR22, miR34a, and miR210 expressions in the same rat mammary carcinoma model. All mentioned results point to phytochemicals as important modulators of miRNAs expression and thus represent important regulators of multi-gene expressions involved in all stages of carcinogenesis [11].

## 4. Materials and Methods

The experiments were approved by the Ethical Commission of the Jessenius Faculty of Medicine of Comenius University (Protocol No. EK1860/2016) and by the State Veterinary and Food Administration of the Slovak Republic (accreditation No. Ro-3239/15-221 and Ro-1640/17-221).

### 4.1. Animals and Induction of Mammary Carcinogenesis, Design of Experiment

Sprague–Dawley female rats (Charles River Laboratories, Sulzfeld, Germany) and BALB/c female mice (Velaz, Prague, Czech Republic) at 5 weeks of age were used in the experiments. Animals were acclimatized to standard vivarium conditions with temperature 23 ± 2 °C, relative humidity –60%, artificial regimen (L/D 12:12 h). During the experiment, rats and mice were fed with the Ssniff^®^ R-Z (M-Z) low-phytoestrogen V1354-0 diet (Soest, Germany) and had an access to drinking water ad libitum. In rats, mammary carcinogenesis was induced by *N*-nitroso-*N*-methylurea (NMU, Sigma, Deisenhofen, Germany) administered intraperitoneally (single dose of 50 mg/kg body weight on average on the 42^nd^ postnatal day). This model mimics high-risk premenopausal women. In mice, a syngeneic model was used; mouse mammary adenocarcinoma 4T1 cells (1 × 10^5^ cells/mouse) were inoculated subcutaneously into the abdominal mammary gland area.

Chemoprevention with dried *C. zeylanicum* (bark, Calendula, Nová Ľubovňa, Slovak Republic; country of origin – Indonesia) began one week before carcinogen administration and lasted 14 weeks after NMU administration in rats. In mice, *C. zeylanicum* administration was initiated on the day of 4T1 cell inoculation and lasted 15 days until the end of the study. In both animal models, *C. zeylanicum* was administered through the diet (powder processed by “cold pelleting procedure”) at two concentrations of 1 g/kg = 0.1% (w/w) and 10 g/kg = 1% (w/w). Rats/mice (*n* = 25/26 per group, total 75/78 animals) were randomly assigned into three experimental groups: (1) control group without chemoprevention/treatment; (2) chemoprevention/treatment with *C. zeylanicum* at a concentration of 0.1% (CIN 0.1); (3) chemoprevention/treatment with *C. zeylanicum* at a concentration of 1% (CIN 1). The rats were weighed and palpated weekly in order to determine the presence, number, location, and size of each palpable tumor. In mice, the growth of tumors was monitored (3 times a week) from the third day after 4T1 cell inoculation and the size of palpated tumors for each mouse was recorded individually. Food intake over 24 h was monitored four times in rats and twice in mice during the experiment. The average daily dose of *C. zeylanicum* per rat was 17.0 mg (CIN 0.1) and 193.7 mg (CIN 1) and 8.1 mg (CIN 0.1) and 8.9 mg (CIN 1) per mice, respectively. In the last week of the animal study, animals were quickly decapitated, the blood from each animal was collected, mammary tumors were excised and the tumor size was recorded.

### 4.2. Histopathological and Immunohistochemical Analysis of Rat and Mouse Tumors

A tissue sample of each rat and mouse adenocarcinoma was routinely formalin-fixed and paraffin-embedded. Rat tumors were classified according to the criteria for the standardized classification of mammary tumors. The additional parameter (grade of invasive carcinomas) was used. Rat tumor samples were divided into low-grade (LG) and high-grade (HG) carcinomas. The criteria for categorization (solidization, cell atypia, mitotic activity index, and necrosis) were chosen according to the standard diagnostic method of classification. HG carcinomas were considered to be tumors with ≥2 positive criteria and LG carcinomas were tumors with ≤1 positive criterion. In addition, mitotic activity index and all tumor areas/necrosis ratio were assessed in mice tumors. Metabolic parameters (total cholesterol, very low-density lipoprotein cholesterol, low-density lipoprotein cholesterol, high-density lipoprotein cholesterol, triacylglycerols, glucose) were evaluated in rat serum using an Olympus AU640 (Olympus Optical, Tokyo, Japan) automatic biochemical analyzer.

The most relevant part of the rat mammary tumor in paraffin block (which includes the typing characteristics and having the largest representation of vital tumor epithelial component, i.e., without regressive changes such as extensive necrosis) was chosen for immunohistochemical analysis. The detection of selected markers for the mechanistic study was carried out by indirect immunohistochemical method on whole paraffin sections, utilizing commercially available rat-specific antibodies (Santa Cruz Biotechnology, Paso Robles, CA, USA; Dako, Glostrup, Denmark; Bioss, Woburn, MA, USA; GeneTex, Irvine, CA, USA; Abcam, Cambridge, MA, USA; Boster Biological Technology, Pleasanton, CA, USA; Thermo Fisher Scientific, Rockford, IL, USA). All steps of the immunohistochemical staining (Autostainer Link 48/Hermes/) were processed according to manufacturers’ recommendations as we described previously. The concentration used for each primary antibody was as follows: caspase-3 1:500, Bax 1:200, Bcl-2 1:200, Ki-67 1:50, VEGFA 1:150, VEGFR-2 1:80, MDA 1:1000, CD24 1:200, CD44 1:200, ALDH1A1 1:500, EpCam 1:160, H3K4m3 1:500, H3K9m3 1:400, H4K20m3 1:300, H4K16ac 1:200. The primary antibodies were visualized by a secondary staining system (EnVision, Dual Link System-HRP, cat. No. K060911, Dako North America, Carpinteria, CA, USA) using diaminobenzidine tetrahydrochloride as a substrate. Negative controls included omission of primary antibody. Immunohistochemically detected antigen expression was evaluated by precise morphometric method. Sections were screened and digital images at magnifications of ×400 were microscopically analyzed (Olympus BX41N). The expression of proteins was quantified as the average percentage of antigen positive area in standard fields (0.5655 mm^2^) of tumor cell hot-spot areas. We analyzed three hot spots per tumor sample using the morphometric method. Morphometric analysis of the digital images was performed using QuickPHOTO MICRO software, version 3.0 (Promicra, Prague, Czech Republic). The values were compared between treated (CIN 0.1 and CIN 1) and non-treated (control) tumor tissue specimens of female rats; at least 60 tumor samples for one marker were analyzed (in total 900 of tumor slides for 15 markers).

### 4.3. miRNA Expression Analysis

Total RNA was isolated from tumor tissues by using a commercially available preparation miRVana microRNA isolation kit (Thermo Fisher Scientific, Waltham, MA, USA). A detailed description of these procedures is available in the supplementary protocol. After the extraction, the RNA was quantified on a NanoDrop ND-2000 Spectrophotometer (Thermo Scientific, Wilmington, DE, USA). Reverse transcription was performed by the TaqMan Advanced miRNA cDNA synthesis Kit (Applied Biosystems, Life Technologies, Carlsbad, CA, USA). The samples of cDNA were stored at −20 °C for future usage. For a quantitative real-time PCR, the miRNA-specific TaqMan™ Advanced miRNA assays Kit (Applied Biosystems Life Technologies, Carlsbad, CA, USA) for tumor-suppressor miR-22, miR-210, miR-34a and for the target oncogenic miR-21 were used. MiR-191-5p was selected as the internal control to normalize the cDNA levels of the samples. Quantitative real-time PCR reaction was performed on an AB7500 Real Time System (Applied Biosystems Life Technologies, Carlsbad, CA, USA). All qPCR reactions were performed in duplicate and Cq values were averaged.

### 4.4. Nucleic Acids Extraction and Bisulfite Conversion

Prior to the isolation of DNA, fresh frozen tumor samples were disrupted by TissueLyser LT (Qiagen, Germany). An average of 50–100 mg of sample and stainless-steel beads 5 mm in diameter (Qiagen, Germany) were added into a precooled tube. Samples were disrupted and homogenized in 200 μL of lysis buffer (Qiagen, Germany) in TissueLyser LT (Qiagen, Germany) at 50 Hz until the tissue was completely disturbed. Homogenized samples were incubated at 56 °C with addition of 20 μL of proteinase K. Genomic DNA was extracted using the DNeasy Blood & Tissue kit (Qiagen, Germany) according to the manufacturer’s protocol. DNA concentration was estimated by the Qubit™ 3.0 Fluorometer (Thermo Fisher Scientific) at a wavelength of 260 nm. At least 50 ng of DNA were used for sodium bisulfite modification using an EpiTect Bisulfite kit (Qiagen, Germany) according to the manufacturer’s recommendation.

### 4.5. Quantitative Methylation Analysis (Pyrosequencing)

Quantitative pyrosequencing was performed with the PyroMark PCR kit (Qiagen, Germany). Predesigned methylation assays were used to determine the methylation status of three CpG sites in the *RASSF1A*, six CpG islands in the *TIMP3*, six CpG islands in the *PTEN*, five CpG areas in the *PITX2,* and four CpG islands in the *ATM* promoter (PyroMark CpG assay, Qiagen, Germany). The total volume of PCR reaction was 25 μL including 20 ng of bisulfite-treated DNA. Thermal cycling protocol included an initial denaturation at 95 °C for 15 min, followed by 45 cycles of amplification: 94 °C for 30 s, 56 °C during 30 s, 72 °C for 30 s, and a final extension at 72 °C for 10 min. The amplification products were confirmed by electrophoresis on 1.75% agarose gel, stained with GelRed Nucleic Acid (Biotinum Inc., Fremont, CA, USA) and visualized on UV transilluminator. Obtained PCR products were analyzed according to the manufacturer’s instructions using the PyroMark Q96 ID System (Qiagen, Germany) with PyroMark Gold Q96 Reagents. Methylation data were evaluated with the instrument software (PyroMark Q96 software version 2.5.8; Qiagen, Germany).

### 4.6. Cell Culture and Experimental Design

The human cancer cell line MCF-7 (human breast adenocarcinoma, ER+, PR+, HER2-) and MDA-MB-231 (human breast adenocarcinoma; ER-, PR-, HER2-) were cultured in Dulbecco’s modified Eagle’s medium with Glutamax-I and Sodium pyruvate (GE Healthcare, Piscataway, NJ, USA) or RPM1 1640 medium (Biosera, Kansas City, MO, USA), respectively. The growth medium was supplemented with a 10% fetal bovine serum (Gibco), 1X HyClone™ Antibiotic/Antimycotic Solution (GE Healthcare) and cells were cultivated in an atmosphere containing 5% CO_2_ in humidified air at 37 °C. Cell viability, estimated by trypan blue exclusion, was greater than 95% before each experiment.

MCF-7 (3 × 10^5^) and MDA-MB-231 (1 × 10^5^) cells were seeded in Petri dishes and cultivated for 24 h in a complete medium with 10% FCS. Cells were treated with EOC (Calendula, Nová Ľubovňa, Slovak Republic) for 24, 48, and 72 h prior to analysis.

### 4.7. Cytotoxicity Assay

The MTS colorimetric assay was used to determine cytotoxic effects of EOC at final dilutions of 1:1250–1:40,000 (MCF-7) or 1:1250–1:160,000 (MDA-MB-231). After 72 h of incubation, 10 µL of MTS (Promega, Madison, WI, USA) was added to each well according to the CellTiter 96^®^ AQueous One Solution Cell Proliferation Assay protocol. After minimum 1 h of incubation, the absorbance was measured at 490 nm using the automated Cytation^TM^ 3 Cell Imaging Multi-Mode Reader (Biotek, Winooski, VT, USA). The absorbance of the control wells was taken as 100% and the results were expressed as a fold of the control. All experiments were performed in triplicate.

### 4.8. 5-Bromo-20-deoxyuridine (BrdU) Cell Proliferation Assay

The BrdU incorporation was used to analyze cell proliferation activity monitored by quantification of BrdU introduced to the genomic DNA during cell growth after EOC treatment (final dilution in range 1:1250–1:40,000 resp. – 1:160,000). DNA synthesis was assessed using colorimetric cell proliferation ELISA assay (Roche Diagnostics GmbH, Mannheim, Germany) following manufacture protocol. The color intensity was measured with Cytation^TM^ 3 Cell Imaging Multi-Mode Reader (Biotek) at 450 nm (reference wavelength: 690 nm). The results were expressed as a fold of the control. All experiments were performed in triplicate. For following analyses, final dilutions (calculated from MTS and BrdU assays) of 1:65000 (MDA-MB-231 cells) or 1:25000 (MCF-7 cells) were used.

### 4.9. Flow Cytometry Analyses Protocol

For flow cytometric analysis (FCM), floating and adherent cells were harvested together 24, 48, and 72 h after treatment (EOC final dilutions 1:25,000 or 1:65,000), washed in PBS, resuspended in PBS, and stained prior to analysis (see table below). Fluorescence was detected after 15–30 min incubation in the dark at room temperature, using a FACSCalibur flow cytometer (Becton Dickinson, San Jose, CA, USA).

^*^ After harvesting, cell suspension is fixed in cold 70% ethanol and kept at −20 °C overnight.

### 4.10. The Examinations of Plant Secondary Metabolites in EOC

The semi-quantitative analysis using the GC-MS was used for evaluation of phytochemical profile of EOC. Agilent Technologies GC 7890A system equipped with a column DB-WAXetr (60 m × 320 µm × 0.25 µm film thickness), with 5975C VL MSED with Triple-Axis detector was used. The method settings were as follows: injector 250 °C, injection volume 0.1 µL, pressure 72.313 kPa; oven program 40 °C for 5 min, then 4 °C/min to 250 °C for 2.5 min, run time 60 min, inlet gas He with average velocity 34.4 cm/sec. MS detector settings: MS source 230 °C, MS Quad 150 °C, solvent delay time 6 min, MS Scan 29–550 *m/z*. The EOC was tested due to the known effects of essential oil (EO) content compounds, wide use of EO in practice, and also the simple accessibility of sufficient amounts of commercially available material. Moreover, the EO content compounds can be very simply quantitatively analyzed using GC-MS.

### 4.11. Statistical Analyses

In the in vivo study, data are expressed as means ± SEM. The Mann–Whitney test, Kruskal–Wallis test, Student’s t-test, and one-way analysis of variance (ANOVA) were the statistical methods used in data evaluation. Tumor volume was calculated according to the formula: V = π. (S_1_)^2^. S_2/_12 (S_1_, S_2_ are tumor diameters; S_1_ < S_2_). In fluorescence assay, ANOVA was first carried out to test the differences between groups; comparisons between individual groups were made using a Student-Newman-Keuls Multiple Comparisons Test. In the in vitro study, data are expressed as means ± SD. Data were analyzed using ANOVA followed by the Bonferroni multiple comparisons test. Differences were considered significant when *p* < 0.05. The quantitative results were calculated from calibration curves, expressed as means ± SD. Data analyses were conducted using GraphPad Prism, version 5.01 (GraphPad Software, La Jolla, CA, USA). The examinations of plant secondary metabolites in the essential oil of *C. zeylanicum* were performed in triplicate.

## 5. Conclusions and Outlook

Plant-based substances are not yet used in the treatment and prevention of breast tumors. In this regard, our study using animal chemopreventive and therapeutic models of BC and an in vitro approach provide original scientific data. *C. zeylanicum* L. showed a significant chemopreventive effect in a model of chemically induced mammary carcinogenesis in rats. Moreover, cinnamon demonstrated a significant therapeutic effect in the 4T1 adenocarcinoma model in mice. The anticancer effect of cinnamon was accompanied by significantly positive changes in the histopathological characteristics of tumors in both rat and mouse models. Immunohistochemical analysis of mammary cancer cells revealed significant proapoptotic, antiproliferative, antiangiogenic, antioxidant, and anti-CSCs effects of cinnamon in vivo. Moreover, pro-apoptotic and antiproliferative effects of cinnamon were confirmed using two human cancer cell lines. In addition, the anticancer efficacy of cinnamon was accompanied by significant positive epigenetic effect in rat tumors. These significant effects of *C. zeylanicum* on numerous molecular markers point to the activation of non-specific signaling linked with mechanisms of anticancer action in our study. This implies the need for immediate attention for their systematic in-depth evaluation in clinical oncology research.

Chemoprevention of BC in humans using plant foods requires the determination of efficacy, the definition of appropriate dosages and undesirable side effects during long-term administration, and method of application, which will require well-designed and controlled clinical trials. The chemopreventive efficacy of selected plant foods (mainly with high antioxidant and epigenetic activities) can also be expected in humans; however, it seems logical that the daily consumption of several different herbs and spices with proved oncostatic activity in vivo (e.g., thyme, oregano, cloves, rosemary, sage, curcuma) will be significantly more efficient than the use of one plant food. The therapeutic efficacy of cinnamon in the 4T1 model is very interesting within cancer research, but there are clear limitations for clinical practice. The 4T1 model represents testing of only one cancer cell line; on the other hand, malignant tumors in humans, whose cells are characterized by broken cell cycle checkpoints and thus intensively proliferate, create conditions for the formation of many mutations, resulting in numerous new tumor lines of different genotypes and phenotypes within the tumor mass. In such malignancies, a highly variable sensitivity not only to conventional therapeutics but also to molecules of natural plant origin can be anticipated.

Innovative clinical strategies that include individual screening programs addressing the needs of high-risk populations, further patient stratification using specific phenotypes and genotypes that are known to be associated with increased BC prevalence, and finally precise diagnosis of BC are key medical approaches that support the change of paradigm in overall BC management from reactive to predictive, preventive, and personalized medicine [95,96]. The use of plant functional foods in the chemoprevention/treatment of BC fits into this concept. Based on the results from our laboratory, specific plant foods appear to be safe and suitable for regular consumption as a part of effective cancer prevention.

## Figures and Tables

**Table 1:** Relative abundance of the major compounds in *C. zeylanicum* essential oil.

No.	Compound	Rt [min]	Relative Content [%]
**1**	**α-Pinene**	9.411	0.926
**2**	**Limonene**	15.969	1.959
**3**	**Eucalyptol**	16.272	2.736
**4**	***o*-Cymol**	18.716	1.657
**5**	**Linalool**	28.069	3.645
**6**	**β-Caryophyllene**	29.704	2.996
**7**	**α-Terpineol**	32.552	1.533
**8**	**Anethole**	36.280	2.418
**9**	**Cinnamaldehyde dimethyl acetal**	40.569	7.181
**10**	**Cinnamaldehyde**	40.959	61.149
**11**	**Cinnamyl acetate**	44.421	6.909
**12**	**Eugenol**	44.753	3.769
**13**	**Benzyl benzoate**	54.749	0.665
	**In Total**	-	97.543

Only compounds exceeding 0.5% of relative content are shown.

**Table 2:** Chemopreventive activity of *C. zeylanicum* in chemically induced rat mammary carcinogenesis at the end of experiment.

Group	CONT	CIN 0.1	CIN 1
Tumor bearing/all animals	23/24	17/24	17/24
Tumor frequency per group *	3.88 ± 0.66	2.46 ± 0.48 (−36.5%)	2.67 ± 0.65 (−31%)
Tumor incidence (%)	95.8	70.8* (−15.5%)	70.8* (−15.5%)
Tumor latency * (days)	73.35 ± 3.83	78.65 ± 3.09 (+5.5 days)	82.76 ± 3.35 (+9.5 days)
Average tumor volume * (cm^3^)	0.54 ± 0.09	0.67 ± 0.16 (+24%)	0.33 ± 0.05# (−39%)
Cumulative tumor volume ** (cm^3^)	48.97	38.22 (−22%)	20.41 (−58.5%)

CONT—control group, CIN 0.1—group with administered *C. zeylanicum* at a concentration of 1 g/kg in diet, CIN 1—group with administered *C. zeylanicum* at a concentration of 10 g/kg in diet. * Data are expressed as mean ± SEM. ** Data are expressed as a sum of volumes per group. Values in brackets are calculated as %-ual deviation from the 100% of non-influenced control group (with exception of latency). Significantly different, * *p* < 0.05 vs. CONT; # *p* < 0.05 vs. CIN 0.1.

**Table 3:** Histopathological characteristics of 4T1 tumors in Balb/c mice after *C. zeylanicum* treatment.

Parameter	CONT	CIN 0.1	CIN 1
**Necrosis/whole tumor area**	5.26 ± 2.13	5.27 ± 0.99	4.89 ± 1.18
**Mitotic activity index**	33.86 ± 2.33	23.96 ± 3.01 *	18.38 ± 1.99 ***

Data are expressed as mean±SEM. Significant difference: * *p* < 0.05, *** *p* < 0.001 vs. CONT.

**Table 4:** The cell cycle distribution in MCF-7 cells after EOC treatment.

**Time (h)**	24		48		72	
**Treatment**	CONT	EOC	CONT	EOC	CONT	EOC
**Sub-G_0_/G_1_**	0.70 ± 0.13	7.73 ± 0.40 *	0.91 ± 0.21	12.45 ± 0.55 *	1.36 ± 0.37	14.66 ± 1.15 **
**G_1_**	68.77 ± 1.47	63.07 ± 1.97 *	69.43 ± 3.68	61.60 ± 0.79 *	70.03 ± 0.34	58.90 ± 2.66 **
**S**	14.30 ± 1.37	12.37 ± 1.27	11.19 ± 2.22	9.59 ± 0.11	10.89 ± 2.08	11.50 ± 1.36
**G_2_/M**	16.23 ± 1.62	16.83 ± 1.23	18.47 ± 0.87	16.35 ± 0.98	17.73 ± 2.51	14.95 ± 1.15

The cell cycle distribution in MCF-7 cells after EOC treatment (1:25,000) was assessed by flow cytometry. Data are expressed as means ± SD of three independent experiments. The significant differences between control and EOC-treated cells were signed as *p* < 0.05 (*), *p* < 0.01 (**).

**Table 5:** The cell cycle distribution in MDA-MB-231 cells after EOC treatment.

**Time (h)**	24		48		72	
**Treatment**	CONT	EOC	CONT	EOC	CONT	EOC
**Sub-G_0_/G_1_**	1.08 ± 0.18	4.20 ± 0.33	0.54 ± 0.12	5.57 ± 0.06	0.70 ± 0.11	6.63 ± 0.09 *
**G_1_**	53.30 ± 2.41	51.60 ± 1.45	58.80 ± 0.33	47.75 ± 0.15	64.83 ± 1.84	47.00 ± 0.20 **
**S**	20.63 ± 1.30	19.35 ± 2.65	19.80 ± 0.85	24.65 ± 0.46	15.27 ± 0.78	25.40 ± 1.37 *
**G_2_/M**	25.00 ± 1.07	24.85 ± 0.15	20.87 ± 1.09	22.05 ± 1.35	19.20 ± 2.25	21.00 ± 2.20

The cell cycle distribution in MDA-MB-231 cells after EOC treatment (1:65,000) was assessed by flow cytometry. Data are expressed as means ± SD of three independent experiments. The significant differences between control and EOC-treated cells were signed as *p* < 0.05 (*), *p* < 0.01 (**).

**Table 6:** Induction of apoptosis in MCF-7 cells after EOC treatment.

**Time (h)**	24		48		72	
**Treatment**	CONT	EOC	CONT	EOC	CONT	EOC
**An^−^/PI^−^**	83.50 ± 1.39	49.60 ± 2.47 **	79.23 ± 1.99	33.10 ± 4.81 ***	80.47 ± 2.17	17.47 ± 1.02 ***
**An^+^/PI^−^**	2.58 ± 0.53	12.77 ± 0.94 *	2.29 ± 0.82	50.67 ± 1.37 ***	2.64 ± 0.69	68.60 ± 0.99 ***
**An^+^/PI^+^**	4.15 ± 0.33	19.00 ± 1.36 *	7.41 ± 1.53	10.34 ± 2.60	5.17 ± 1.19	5.68 ± 0.51
**An^−^/PI^+^**	9.77 ± 1.53	18.64 ± 0.87 *	11.06 ± 1.07	5.89 ± 1.03	11.74 ± 1.05	8.24 ± 0.78

Induction of apoptosis in MCF-7 cells after EOC treatment (1:25,000) was analyzed after Annexin V and PI staining protocol for flow cytometry. The percentage of events in the non-apoptotic (lower left, An−/PI−), early apoptotic (lower right, An+/PI−), late apoptotic (upper right, An+/PI+), and necrotic (upper left, An−/PI+) quadrants is indicated. Values are the means ± SD of three independent experiments. The significant differences between control and EOC-treated cells were marked as *p* < 0.05 (*), *p* < 0.01 (**), *p* < 0.001 (***).

**Table 7:** Induction of apoptosis in MDA-MB-231 cells after EOC treatment.

**Time (h)**	24		48		72	
**Treatment**	CONT	EOC	CONT	EOC	CONT	EOC
**An^−^/PI^−^**	90.00 ± 0.90	43.10 ± 2.23 ***	92.27 ± 1.40	44.00 ± 3.10 ***	95.00 ± 2.34	26.65 ± 3.22 ***
**An^+^/PI^−^**	4.23 ± 0.24	15.35 ± 1.31 *	1.58 ± 0.44	18.10 ± 1.10 **	1.14 ± 0.19	35.25 ± 3.08 **
**An^+^/PI^+^**	2.05 ± 0.54	24.50 ± 3.04 **	2.18 ± 0.06	20.75 ± 1.55 **	1.22 ± 0.18	19.60 ± 1.00 **
**An^−^/PI^+^**	3.76 ± 0.20	17.05 ± 1.25 *	3.98 ± 0.24	17.15 ± 0.45 *	2.67 ± 0.05	18.50 ± 0.80 *

Induction of apoptosis in MDA-MB-231 cells after EOC treatment (1:65,000) was analyzed after Annexin V and PI staining protocol for flow cytometry. The percentage of events in the non-apoptotic (lower left, An−/PI−), early apoptotic (lower right, An+/PI−), late apoptotic (upper right, An+/PI+), and necrotic (upper left, An−/PI+) quadrants is indicated. Values are the means ± SD of three independent experiments. The significant differences between control and EOC-treated cells were marked as *p* < 0.05 (*), *p* < 0.01 (**), *p* < 0.001 (***).

## Supplementary Materials

Supplementary materials can be found at https://www.thermofisher.com/document-connect/document-connect.html?url=https%3A%2F%2Fassets.thermofisher.com%2FTFS-Assets%2FLSG%2Fmanuals%2Fcms_055423.pdf&title=bWlyVmFuYSZ0cmFkZTsgbWlSTkEgSXNvbGF0aW9uIEtpdCAoRW5nbGlzaCAp.
